# The Trajectory of Revisional Bariatric Surgery: Open to Laparoscopic to Robotic

**DOI:** 10.3390/jcm13071878

**Published:** 2024-03-25

**Authors:** Noura Jawhar, Jack W. Sample, Marita Salame, Katie Marrero, Daniel Tomey, Suraj Puvvadi, Omar M. Ghanem

**Affiliations:** 1 Department of Surgery, Mayo Clinic, Rochester, MN 55905, USA; jawhar.noura@mayo.edu (N.J.);; 2 Carle Foundation Hospital General Surgery Residency, Champaign, IL 61801, USA; 3 Department of General Surgery, Houston Methodist Hospital, Houston, TX 77030, USA; 4 College of Health Solutions, Arizona State University, Phoenix, AZ 85004, USA

**Keywords:** obesity, metabolic and bariatric surgery, revisional bariatric surgery, laparoscopic, robotic-assisted

## Abstract

Metabolic and bariatric surgery (MBS) is the most effective therapeutic intervention for patients with obesity, with sleeve gastrectomy (SG) being the most commonly performed primary MBS procedure. Long-term studies have demonstrated that 15–20% of patients require revisional bariatric surgery (RBS) due to weight-related issues or surgical complications. Despite the gold standard being laparoscopic revision, there are other available approaches such as open or robotic-assisted. An extensive literature review was performed for articles from their inception to February 2024. A descriptive review of MBS procedures (SG, Roux-en-Y gastric bypass (RYGB), single anastomosis duodeno-ileostomy (SADI) and biliopancreatic diversion-duodenal switch (BPD-DS)) was carried out to report and compare outcomes between primary and revisional bariatric surgery. A similar review was conducted to compare outcomes of revisional approaches (open, laparoscopic, robotic). RYGB remains the dominant RBS with a similar safety profile compared to revisional SADI and BPD-DS. In terms of the RBS surgical approach, all three options showed comparable short and long-term outcomes, with robotic RBS being associated with longer operative time and variable length of stay. Additional long-term studies are required to further validate our conclusions.

## 1. Introduction

Obesity is a chronic and endemic disease with increasing prevalence worldwide. Metabolic and bariatric surgery (MBS) offers a safe and effective long-term method to achieve weight loss and obesity-related medical condition improvement and resolution [1]. The number of performed MBS procedures has increased exponentially in parallel with the global rise in obesity. Sleeve gastrectomy (SG) and Roux-en-Y gastric bypass (RYGB) remain the most commonly performed primary MBS procedures [2]. Despite the well-known long-term success of MBS, a certain number of patients require revisional surgery following primary MBS procedures due to insufficient weight loss, weight recurrence and surgical complications [3]. Indeed, revisional bariatric surgery (RBS) now represents 16.7% of all bariatric procedures performed in 2019, with an overall increase of 60% in performed RBS procedures in the USA between 2011 and 2019 [4].

Despite advancements within the bariatric surgical field, RBS remains a surgical challenge due to the altered anatomy and the presence of significant adhesions. Recent reports have demonstrated an increased incidence of 30-day complications, higher risk of perioperative morbidity and mortality, longer operative time and inconsistent long-term results associated with RBS compared to primary MBS procedures [5,6,7]. RBS has been categorized by Brethauer et al. as either conversion, corrective, or reversal [8]. Corrective procedures include preserving the anatomy of the primary MBS procedure and re-exploring the anatomy to overcome existing postoperative complications. Conversions include rearranging the anatomy to perform a different bariatric procedure and reversals imply the restoration of the original anatomy [5,9]. Corrective RBS is indicated mainly for the management of chronic postoperative complications, such as marginal ulcer or anastomotic stricture. Conversions are commonly performed due to insufficient weight loss, weight recurrence, or recurrence of obesity-related medical conditions such as gastroesophageal reflux disease (GERD). Reversals are typically a last resort RBS option for patients with severe nutritional and metabolic deficiencies or excessive weight loss [10]. 

Traditionally, most reoperations are performed with an open approach, especially when the primary MBS procedure was open. However, with technological advancements offering a magnified view of the surgical field and allowing for the use of finer instruments, the laparoscopic technique has been widely embraced, with data showing shorter recovery time and decreased postoperative pain. The laparoscopic approach now represents the standard surgical approach, with the robotic-assisted approach as a recent and effective alternative in both MBS and RBS [5,11,12,13]. The utilization of such technology is advantageous in dealing with complex surgical anatomy and can overcome difficulties typically encountered in laparoscopic surgery. However, there is a scarcity in the current literature demonstrating the long-term efficiency and role of a robotic-assisted approach in MBS and RBS [11]. Zhang et al. reported that while robotic bariatric surgery was associated with longer operative time, there was a similar safety and efficacy profile compared to laparoscopic bariatric surgery [14].

Despite these challenges, the number of revisional procedures is expected to rise in parallel with the rise in MBS procedures [5,6,7]. The aim of this review is to report the indications for RBS following the most common primary MBS procedures, investigate the outcomes of revisional procedures compared to primary MBS procedures and explore the outcomes of the different revisional approaches (open, laparoscopic and robotic). 

## 2. Indications for Revisional Bariatric Surgery

*Sleeve Gastrectomy (SG)*

SG is the most commonly performed bariatric procedure worldwide since 2015, with laparoscopy being the gold standard surgical approach [2,15]. Laparoscopic sleeve gastrectomy (Lap SG) has emerged as a popular choice among bariatric surgeons, as it is technically simpler than the traditional RYGB and has demonstrated favorable short and long-term outcomes. 

Lap SG has several notable advantages, which include reduced operative time, perceived technical feasibility and excellent weight loss outcomes [16]. Despite these advantages, Lap SG is associated with several potential drawbacks. Known potential complications include weight recurrence [17], GERD, sleeve stenosis and staple line leak [16]. The management of these complications is broad and often multidisciplinary but may ultimately require revisional bariatric surgery. 

### 2.1. Indications for Revision after Sleeve Gastrectomy 

#### 2.1.1. Weight-Related Issues (IWL and WR)

SG has demonstrated excellent excess weight loss (%EWL) results exceeding 50% at 5-year follow-up [18]. Despite these results, studies have begun to show surprisingly considerable rates of weight recurrence with long-term follow-up, with rates as high as 70% at 6 years and 80% at 10 years [17,19]. 

Weight-related complications after primary SG are classified as insufficient weight loss (IWL) or weight recurrence (WR). The definitions for IWL and WR lack uniformity, resulting in varied outcomes within the literature [20]. IWL is loosely defined as less than 50% estimated weight loss (EWL) at 18 months after MBS. WR is generally defined as undesired weight gain after MBS resulting in a body mass index (BMI) greater than 35 kg/m^2^ or an increase in weight exceeding 10 kg from the nadir body weight after MBS with recurrence of comorbidities [21]. IWL or WR are frequently identified as the most common indication for revision after Lap SG [19,22,23]. 

The etiology of WR following Lap SG is likely multifactorial. Proposed mechanisms include the creation of a large-volume sleeve gastrectomy, dilatation of the sleeve, incomplete removal of the gastric fundus, residual antrum and postoperative lifestyle factors [20]. Studies have shown a significant association between increased sleeve volume and rates of WR [24]. Retained fundal tissue due to incomplete resection of the gastric fundus permits continued release of appetite-stimulating ghrelin, hindering the metabolic weight loss mechanism of Lap SG. A retrospective review conducted by Obeidat demonstrated that patients with an antrum-remnant divided 6 cm from the pylorus were more likely to experience WR compared with those divided at 2 cm from the pylorus [25]. Additionally, routine postoperative follow-up and lifestyle behavioral adaptations, or lack thereof, may influence the likelihood of WR following Lap SG [20,26,27,28]. 

Some studies suggest that WR is more commonly seen in restrictive bariatric procedures, such as SG [29]. When revision is indicated for WR after Lap SG, various bariatric procedures can be performed to restimulate weight loss, including re-sleeve gastrectomy, conversion to RYGB, or conversion to biliopancreatic diversion with duodenal switch (BPD-DS). SG was first performed as the first part of the two-stage BPD-DS surgery. Thus, conversion to BPD-DS is an appropriate choice for patients with weight-loss failure after Lap SG. RYGB is also a suitable option, especially in patients with concomitant GERD [29,30].

#### 2.1.2. Gastroesophageal Reflux Disease (GERD)

Being a stomach restricting procedure by mechanism, SG has been implicated as a risk factor for developing GERD. The creation of a gastric sleeve reduces gastric volume while increasing intraluminal pressure, which may allow easier reflux of gastric contents. Furthermore, SG may alter the angle of His or disrupt the LES, which can influence the development of reflux [31,32]. 

Once a diagnosis has been made, dietary and lifestyle modifications and acid reduction therapy, typically with proton pump inhibitors (PPIs), are employed as first-line therapy. Patients who fail medical management may be candidates for surgical revision, in which conversion to RYGB is considered the gold standard. RYGB is effective at treating reflux by altering gastrointestinal anatomy to reduce esophageal exposure and bile reflux. Casillas and colleagues found conversion to RYGB effective in improving GERD symptoms in 97% of patients [33].

#### 2.1.3. Sleeve Stenosis

Sleeve stenosis is one of the most common complications following Lap SG. The incidence of SG stenosis has been reported to be 0.1–4%; however, some authors suggest that the true incidence is much higher due to underdiagnosing [34,35,36]. SG stenosis can clinically manifest as early satiety, nausea, dysphagia and rapid weight loss after Lap SG. In severe cases of stenosis, the increased proximal luminal pressure can cause staple line leak. 

Sleeve stenosis is often categorized as anatomic or functional, with the former being much more commonly reported. Anatomic stenosis may be caused by technical aspects of the operation, for example, oversewing of the staple line, improper stapler angulation, or using an incorrectly sized bougie [34,35]. Functional stenosis is less common and thought to be secondary to factors that disrupt the sleeve architecture, such as a misaligned staple line, which can result in transient, or functional, sleeve stenosis [34,35,37]. 

The diagnosis and early management of SG stenosis is typically endoscopic. If endoscopic management fails to improve symptoms, patients may require revisional surgery. The most common secondary surgery for stenosis after Lap SG is conversion to RYGB, performed by creating a gastric pouch proximal to the area of stenosis. Other surgical revision options include seromyotomy and gastric wedge resection of the stenotic area [36,38]. These procedures can offer successful resolution of stenosis but are associated with higher complication rates compared with RYGB [38]. 

#### 2.1.4. Staple Line Leak

One of the most serious complications following bariatric surgery, staple line leak, carries significant morbidity and the possibility of mortality. The overall incidence of staple line leak following Lap SG is estimated to be 1–2% and is often classified based on the timing of leak relative to surgery [39,40]. The degree of clinical manifestations due to a staple line leak are variable and often influence the approach to management. 

There are several factors thought to play a role in the cause of staple line leak after Lap SG. Firstly, underlying patient factors and comorbidities may influence the acute wound healing process, such as smoking, diabetes mellitus, and infection. Improper tissue handling, incorrect bougie size, and the degree of tension when firing the stapler are also factors that may influence the risk of leak. Interestingly, most leaks after Lap SG occur along the proximal staple line near the gastroesophageal junction, suggesting an anatomic consideration that may influence leak [40]. 

Early recognition of staple line leak is paramount to management. Endoscopic therapy is the gold standard for management of staple line leak after SG. Many endoscopic procedures exist to treat staple line leak, including deployed sealants, stenting, or clips. However, the efficacy of these nonsurgical techniques remains unclear. In some scenarios, healthy and stable patients can be managed conservatively with antibiotics, acid reducing medications and drain placement. Revisional bariatric surgery is usually reserved for severe cases, typically those that are chronic or have exhausted the limitations of endoscopic therapies. Revisional surgery for staple line leak after SG often requires the removal of the faulty staple line with creation of a new anastomosis at an area of healthy tissue. Staple line leak cases need to be managed earlier rather than later since proximal GI leaks can potentially lead to further complications such as gastrobronchial fistula [41]. The available surgical options are Roux-en-Y esophagojejunostomy (RYEJ) or fistulojejunostomy. RYEJ has a low rate of leak recurrence; however, it is associated with well-established complications related to the new surgical anatomy, such as reflux and nutritional deficiencies [40].

*Roux-en-Y gastric bypass (RYGB)*

RYGB is a widely prevalent bariatric intervention, standing as the second most commonly performed procedure in the United States, following SG [2]. Despite being regarded as a safe choice with proven short, mid and long-term efficacy in achieving significant weight loss and resolving obesity-related medical conditions, RYGB is not without its complications. Risks associated with this primary MBS procedure include the potential development of long-term complications such as marginal ulcers (MUs) and anastomotic strictures, weight recurrence and postprandial hypoglycemia [42], which tend to require revisional surgery as an effective treatment method. Available RBS options (corrective, conversion, reversal) following a failed primary RYGB include pouch resizing with/without revision of the gastrojejunal anastomosis (GJA), distalizing RYGB (DRYGB), conversion to Lap SG or BPD-DS, or complete reversal of the initial RYGB [43]. 

### 2.2. Indications for Revision after Roux-en-Y Gastric Bypass

#### 2.2.1. Marginal Ulcer (MU)

MU is a common complication following RYGB, typically developing at or near the gastrojejunal (GJ) anastomosis, with the majority occurring within the initial year after the surgery. The reported incidence rate of MU displays considerable variability, ranging from 0.6% to 25% [44,45]. Despite the uncertainty surrounding its exact etiology, several potential risk factors have been identified, including diabetes, *Helicobacter pylori* infection, tobacco use, alcohol consumption and nonsteroidal anti-inflammatory drug (NSAID) use, among others [46]. Symptoms of MU can manifest differently, ranging from asymptomatic cases to patients experiencing epigastric pain, nausea/vomiting, or gastrointestinal bleeding. This diversity in symptoms could contribute to the observed variations in reported incidence rates [46]. 

Upper endoscopy is considered as the gold standard in clinical practice for diagnosing MU post-RYGB. Endoscopic examination allows the identification of single or multiple ulcers. Biopsies performed during endoscopy help exclude alternative causes of symptoms and confirm the diagnosis [47]. A computerized tomography (CT) scan can also be commonly employed in outpatient or emergency cases, providing valuable insights and facilitating early detection [48].

The initial MU management includes addressing modifiable risk factors like smoking, NSAID use and alcohol, combined with the administration of proton-pump inhibitors and sucralfate. However, when medical treatment proves insufficient for refractory or recurring cases, or in the presence of associated gastrogastric fistulas, revisional surgery should be considered [49]. Surgeons commonly recommend removing ulcerated GJ anastomoses and restoring the integrity through hand-sewn or stapled reanastomosis for refractory or ischemic MUs, with vagotomy considered if acidity is implicated [44,49,50]. 

#### 2.2.2. Anastomotic Stricture

Anastomotic strictures are a relatively common complication following RYGB, with reported incidence rates ranging from 0.8 to 27%. Such rates include both early and late postoperative periods. Most strictures typically occur early following RYGB, with incidence rates of 0.8–5% [51,52,53,54]. These strictures most commonly occur at two sites: the jejunojejunostomy and the gastrojejunostomy, with the latter being the primary location of occurrence. Symptoms often include persistent or worsening postprandial vomiting, usually accompanied by pain, leading to dysphagia and malnutrition in severe cases [55]. 

The etiology of stricture formation is multifactorial and involves factors such as local tissue ischemia, anastomotic tension, recurrent marginal ulceration and surgical technique, among others [56]. Numerous studies have explored factors associated with GJ stenosis in patients undergoing RYGB, with evidence suggesting that hand-sewn construction of the gastrojejunostomy can reduce the incidence of strictures [55,57]. Multiple studies have shown that circular-stapled anastomoses, especially the 21 mm stapler size, result in significantly higher occurrence rates of stricture compared to linear-stapled and hand-sewn techniques [54,58,59]. 

Early investigation with upper endoscopy is essential for patients who present with upper gastrointestinal symptoms post-RYGB to assess the presence of a GJ stricture [52]. If identified, immediate intervention is recommended, utilizing flexible endoscopy combined with pneumatic balloon dilation to achieve a safe maximal diameter as well as lumen stents following failed endoscopic treatments [60]. Studies have demonstrated the effectiveness of this approach in managing early GJ strictures after RYGB, with successful responses reported in 17% to 67% of cases following the initial dilation, and 3% to 8% requiring three or more dilations [53,55]. Late strictures, however, exhibit reduced responsiveness to endoscopic dilation, often necessitating revisional surgery. While the common approach in such cases involves laparoscopic revisions, it is worth noting that the procedure may pose technical challenges [53]. 

#### 2.2.3. Weight-Related Issues (IWL and WR)

IWL and WR are recognized challenges that can occur after RYGB, leading to the recurrence of obesity-related medical conditions and a decline in the quality of life [61]. Patients may experience a plateau in weight loss or, over time, regain some of the weight initially lost. Despite RYGB’s effectiveness in achieving significant weight loss, long-term success can be impeded by various factors, including anatomical and technical aspects, genetic predisposition, psychological factors and behavioral determinants, among others. Anatomical complications including the enlargement of the GJ stoma diameter, dilation of the gastric pouch (greater than 5 cm in diameter) and the presence of a gastrogastric fistula are associated with WR following RYGB [62]. 

Patients achieving significant initial weight loss may later experience substantial weight recurrence, with rates reported at 36.9% after approximately 6.9 years post-surgery [63]. Another study by King et al. concluded that the largest and most significant change in weight regain occurred 2 years after reaching the lowest weight and continued to increase 5 years following RYGB. The study suggested that 50.2% of patients experienced ≥15% weight regain 5 years after reaching the nadir [61]. The actual prevalence remains unclear because there is no consensus on the definition for significant weight recurrence. 

The management of weight relapse involves multiple strategies. Select cases may benefit from obesity pharmacotherapies and endoscopic bariatric therapy [43,64,65]. Endoscopic therapies such as transoral outlet reduction (TORe) have become a popular treatment option for WR management due to the feasibility of the endoscopic platform in reducing the GJ anastomosis diameter and gastric pouch volume. This mechanical restriction created by full-thickness endoscopic suturing also allows for adjustment in dietary behaviors by decreasing hunger and improving satiety [43,65]. Surgical revision, accounting for 16.8% of all bariatric operations in 2019, reflects its popularity [2]. While revisional surgery is considered a safe and viable option for managing weight recurrence, it is less effective than primary procedures and has been associated with postoperative adverse events [66,67]. Common revisional surgery options for WR following RYGB include pouch resizing with/without revision of the GJA, DRYGB with/without pouch/GJA resizing, conversion to duodenal switch (BPD-DS or SADI-S), conversion to single anastomosis jejunal-ileal bypass (SAJI), or complete RYGB reversal [68]. While surgical revision is effective for inducing weight loss in the short and mid-term, there is a risk of long-term complications such as severe protein malnutrition, which would require further revision [69]. Conversion to BPD-DS or SADI-S has been shown to provide promising long-term weight-loss outcomes; however, due to technical difficulties and the association of early and late complications, it is not commonly performed [70].

#### 2.2.4. Postprandial Hypoglycemia (PPH)

PPH is a long-term complication of RYGB that is often unrecognized and difficult to diagnose. It typically occurs within the first or second postoperative year with an incidence rate of 0.1–34% [71]. Symptoms of PPH include confusion, altered levels of consciousness, weakness, diaphoresis and possible visual disturbances after a meal rich in carbohydrates. Symptoms are more pronounced with stress and physical activity [71,72].

The incomplete understanding of the underlying pathophysiology of PPH and variable patient presentation make the management of PPH challenging [71]. Initial management typically includes dietary and lifestyle modifications, such as adhering to frequent small meals high in protein and fiber, and pharmacologic therapy, including acarbose, nifedipine, octreotide and recent use of glucagon-like peptide 1 (GLP-1) receptor antagonists [72]. The majority of patients are effectively treated with dietary modification alone. In refractory cases, gastrostomy tube placement, revision/restriction of the gastrojejunostomy, or reversal of the RYGB may be necessary [73]. Proposed mechanisms of PPH suggest that accelerated passage of food in the Roux limb stimulates increased secretion of incretion, which elevates postprandial levels of insulin and GLP-1. The reversal of the RYGB anatomy would restore normal food passage and thus alleviate treatment-resistant PPH [71,72,73]. Further studies are required to fully understand the etiology of PPH and refine treatment options in the long term.

## 3. Outcomes of Primary Bariatric Surgery versus Revisional Surgery

Compared to primary MBS, RBS is considered technically challenging due to the manipulated anatomy and presence of adhesions. As a result of this, the literature has shown that operative time and the rates of intra-operative and postoperative complications are significantly higher than primary MBS. 

Pedziwiatr et al. conducted a meta-analysis and found that revisional RYGB was associated with inferior short and long-term outcomes compared to the respective primary RYGB. They reported that revisional RYGB had a significantly higher complication rate, increased mortality rate and less substantial weight loss. No significant difference was observed in the resolution of obesity-related medical conditions between the two RYGB groups [74]. Mahawar et al. reported a greater risk of postoperative complications following revisional RYGB (29.5% vs. 13.9%) and revisional SG (10.5% vs. 5.2%) when compared to their respective primary MBS procedures. The revisional RYGB group had a higher mortality rate compared to primary RYGB (1.3% vs. 0.2%), which was not observed when comparing revisional SG to primary SG (0% vs. 0.1%) [67]. Revisional SG (conversion to SG) was reported to have a higher reoperation rate (4.8% vs. 1.6%) compared to the primary SG group; this was not found among the primary and revisional RYGB groups, as they had comparable rates (8.6% vs. 8.4%). Another significant variable noted was the increased leak rate observed in the revisional RYGB group (5.8% vs. 1.0%) when compared to the primary RYGB group. The study was in agreement with the literature and showed increased mean operative time (201.6 min for the revisional RYGB group vs. 127 min for the primary RYGB group, 133.2 min for the revisional SG group vs. 106 min for the primary SG group) and increased mean length of stay (5.8 days for the revisional RYGB group vs. 4.5 days for the primary RYGB group, 3.8 days for the revisional SG group vs. 3.6 days for the primary SG group) [67]. 

Zhang et al. performed a matched comparative analysis and reported that revisional RYGB resulted in increased conversions to open surgery, higher intraoperative bleeding (463.7 mL vs. 133.3 mL), greater likelihood of admission to the intensive care unit (14% vs. 1%), higher 30-day reoperation rate (9% vs. 2%), longer operative time (272.5 min vs. 175.5 min), longer length of stay (5.6 days vs. 2.5 days), higher readmission rate (16% vs. 7%) and greater incidence of intraoperative (8% vs. 1%) and postoperative complications (55% vs. 28%) compared to primary RYGB [75]. It was also found that revisional RYGB led to a significantly lower percentage of weight loss at 1 year (27% vs. 37%), indicating that primary RYGB provides sustained weight loss in comparison. There was no significant difference in the 30-day mortality rate between the two groups [75]. Similar results were presented by Vallois et al. in a more recent matched analysis; again, revisional RYGB resulted in a significantly lower excess weight loss at 1 year compared to primary RYGB. When comparing weight loss outcomes, it was revealed that patients who underwent primary SG experienced superior and sustainable weight loss compared to patients who underwent a conversion to SG (75.9% vs. 62.6%, *p* = 0.008) [76]. Iranmanesh et al. reported significantly longer operative times (203 min vs. 154 min, *p* < 0.001), increased readmission rate for oral intolerance (10.5% vs. 6.7%, *p* = 0.046) and increased risk of gastrojejunal stricture (6.4% vs. 2.7%, *p* = 0.013) were found in the revisional robotic RYGB group compared to the primary robotic RYGB group. However, there were no significant differences in overall complications, anastomotic leak, conversion, or reoperation rates [77]. 

In the discussion on RBS, single anastomosis duodeno-ileostomy (SADI) and BPD-DS are typically surgeries that patients are revised to. Therefore, the literature in looking at SADI and BPD-DS revisions themselves is much scarcer. Bennett et al. reported that revisional DS procedures were associated with higher risk of SSI, increased reoperation rate and greater readmissions [78]. The study reached the conclusion that, similar to the superior profile of SG in the field of MBS, revisional SG/conversion to SG demonstrates an improved early postoperative safety profile and superior outcomes in comparison to revisional RYGB and DS [78]. 

Most comparison studies concluded that the robotic approach is a safe alternative with similar postoperative morbidity, longer operative time and higher costs when compared to the laparoscopic approach. However, several studies did report decreased incidence of anastomotic leak, conversion to open surgery, postoperative bleeding and stricture rates [79,80,81,82]. 

Although most of the studies investigating comparing MBS and RBS are retrospective in nature with differing patient populations and numbers, they all present similar outcomes of increased perioperative complications associated with RBS procedures. Despite these risks, the indications for RBS and therapeutic benefits achieved by RBS with weight loss, improvement in surgical complications and remission of obesity-related medical conditions outweigh the risks and potential negative postoperative outcomes. 

## 4. Open versus Laparoscopic Approach

There is no clear standardized surgical approach or procedure in revisional surgery. While the laparoscopic approach has been deemed as the most frequently utilized surgical technique for bariatric surgery, open revisional procedures are still performed. Shimizu et al. reported significant differences in postoperative complications when comparing open revisional surgery to laparoscopic revisional surgery. The open approach was found to have a significant increased length of stay (9.5 days vs. 5.4 days, *p* = 0.03), increased intraoperative bleeding (246.2 mL vs. 122.1 mL, *p* < 0.01), greater risk of early complications (39.4% vs. 15.7%) and higher rate of minor complications (30.3% vs. 13.2%) [83]. Buchs et al. compared the postoperative outcomes after open and laparoscopic revisional RYGB. Despite the popularity of the laparoscopic approach, open revisional RYGB was associated with decreased postoperative complications (10.7% vs. 14.3%) and a shorter operative time (250 min vs. 270 min). Laparoscopic revisional RYGB was found to have a shorter length of hospital stay (8 days vs. 9 days) and a lower late reoperation rate (19% vs. 25%) when compared to the open group [6] (Table 1).

Other than the above-mentioned studies, there is a scarcity in the current literature comparing open revisional surgery and laparoscopic revisional surgery. However, the results from above indicate that the laparoscopic approach is associated with an optimal safety and efficacy profile in MBS and RBS procedures. 

## 5. Laparoscopic versus Robotic Approach

The minimally invasive approach has become the standard of care in MBS, with laparoscopic surgery being the gold standard for primary bariatric surgery. Due to surgical advancements and the introduction of innovative technology, the robotic approach has been deemed as an advantageous alternative approach for complex procedures. Laparoscopic surgery has its own limitations of two-dimensional visualization, rigid instruments and poor ergonomics. These limitations are prominent in RBS when encountering a complex anatomy and intense adhesions. The robotic approach could overcome such limitations with improved ergonomics, improved camera control, three-dimensional visualization and flexible endowristed instruments [11,12,84].

King et al. performed a retrospective analysis comparing the laparoscopic approach versus the robotic approach in RBS. They reported that the robotic approach had a decreased rate of 30-day major complications with no significant differences in minor complications, intraoperative complications, or readmission rates when compared to the laparoscopic approach [85]. While King et al. did not encounter any conversions to open surgery, Nasser et al. concluded that the robotic approach of RBS was associated with a higher incidence of conversion to open surgery (0.5%) compared to the laparoscopic approach [86]. This could be explained by varying levels of surgeon expertise and surgeon comfort. It is also possible that the institution’s nature could play a role, as a high-volume robotic center may have a lower conversion risk due to the training and preparedness of the surgical staff. The learning curve of the institution and the surgical teams’ training could also be correlated with operative time. The consensus among the literature is that the robotic approach is associated with a longer operative time, while the length of hospital stay is inconsistent and conflicting, with some studies reporting a shorter hospital stay [6,87] while others reported a longer stay [85,86]. A recent analysis of 26,404 revision cases from the MBSAQIP database found longer operative time (119.5 vs. 173.7 min, *p* < 0.0001) and length of stay (1.9 vs. 2.3 days, *p* = 0.0002) with the robotic approach [88].

Buchs et al. compared the postoperative outcomes after revisional laparoscopic and revisional robotic RYGB. The robotic group was associated with a lower risk of postoperative complications (0% vs. 14.3%), decreased open conversion rate (0% vs. 14.3%), shorter length of hospital stay (6 days vs. 8 days) and longer operative time (352 min vs. 270 min) [6]. Beckmann et al. performed a similar analysis and found a shorter length of stay (4.9 vs. 6.2 days) and fewer postoperative complications (7.3% vs. 22.2%) with the revisional robotic RYGB group [87]. 

Acevedo et al. further compared revisional laparoscopic RYGB and revisional robotic RYGB and reported a higher rate of postoperative sepsis in the robotic group when compared to the laparoscopic group (1.0% vs. 0%, *p* = 0.04). Within the same study, it was found that revisional laparoscopic RYGB had a higher rate of transfusion requirements compared to revisional robotic RYGB (2.9% vs. 0.6%, *p* = 0.02) [88]. Nasser et al. also reported that revisional robotic RYGB had significantly lower risk of perioperative morbidity with lower rates of respiratory complications, surgical site infection and postoperative bleeding requiring transfusion than revisional laparoscopic RYGB [86]. When comparing revisional laparoscopic SG and revisional robotic SG, Nasser et al. demonstrated an increased risk of SSI and sepsis in the robotic group. A significantly higher reoperation rate was also reported in the revisional robotic SG group [86]. Further studies are required in order to fully differentiate the variability in surgeon technique and experience to truly determine what factors are contributing to such differences. 

In exploring the MBSAQIP database, Clapp et al. reported that the laparoscopic approach of RBS and robotic approach of RBS were equivalent in overall postoperative complication rates. The only significant differences noted between the two groups were the operative time and length of stay, which were both increased in the robotic group. They concluded that the robotic approach is as safe as the well-known laparoscopic approach [89]. The recent systematic review and meta-analysis by Bertoni et al. showed comparable results in comparing postoperative complications, conversions, length of stay and operative time between the laparoscopic and robotic approach, concluding that while the robotic RBS had no significant advantage over laparoscopic RBS in terms of those variables, the robotic approach did show an equivalent safety and efficacy profile [7]. Moon et al. also presented similar findings, reporting no difference between the laparoscopic RBS and robotic RBS groups in the mean length of hospital stay, 30-day readmission rate, or 30-day reoperation rate. The results remained similar when the baseline difference in body mass index was accounted for [90] (Table 2).

Despite the documented longer operative time in most of the literature, the robotic approach could be proposed as an alternative to the laparoscopic approach due to the comparable risk of postoperative complications experienced in both approaches. 

## 6. The Evolution of Robotic Surgery and the Learning Curve

Due to the complex surgical anatomy and extensive adhesiolysis and intensive dissection required, robotic-assisted RBS is an appealing approach. Despite the novel inclusion of robotic training in general surgery residency and fellowship curriculums, the literature on robotic RBS is limited. Only a few reports demonstrate the effectiveness of robotics for revisional surgery with overall complication rates of 0–17% along with acceptable postoperative weight loss [6,13,91]. Several studies have focused on the learning curve of robotic MBS procedures. Although a specific number of cases required has not been established, the literature has revealed a faster-paced learning curve for the robotic platform when compared to the conventional laparoscopy. Buchs et al. reported that the robotic RYGB learning curve was around 15 cases compared to the 75–100 cases for laparoscopic RYGB [92,93], with a significant decline in operative time of robotic RYGB and surgical mastery after the completion of 14 cases [94]. Vilallonga et al. demonstrated 20 cases as the learning curve for robotic SG and showed a decrease in operative time once this number has been achieved [95]. Romero et al. established that once the initial 25 cases of robotic SG were performed, a trend in decreasing operative time was observed [96]. 

Despite the growing demand and interest for the robotic approach and the shorter learning curve, operative times remain shorter and favorable with the laparoscopic approach. Longer operative times associated with the robotic technique could be explained by the time to properly dock the robot and the use of hand sewing techniques versus the common use of stapling anastomosis techniques observed in laparoscopic procedures [9,13]. The use of ergonomic and articulated instruments with a three-dimensional visualization and multi-quadrant access of the surgical field allows for fine dissection, proper handling of fragile tissue and hand-sewn anastomoses. This could result in improved postoperative outcomes when compared to the laparoscopic approach. Several studies have reported that despite the longer operative time and cost of surgical training, the robotic approach offers lower anastomotic leak rates compared to the laparoscopic approach [8,9,13,79,97]. The challenge of operating in a hostile environment with abdominal wall thickness, excessive visceral fat, modified tissue planes and extensive adhesions explains the risk of such leaks. Snyder et al. reported a significant difference in the incidence rate of GJ anastomotic leaks between the laparoscopic stapler group and robotic hand-sewn group (1.7% vs. 0%, *p* = 0.04) [79]. The meta-analysis by Li et al. also found that while there were no significant differences in the overall postoperative complications, the reoperation rate, conversion rate, mortality rate, or length of stay, the robotic approach had a lower incidence of anastomotic leak [98]. This suggests that the robotic approach could be a beneficial alternative to the laparoscopic approach in the RBS field and should be advocated for with proper training.

Operative time depends on the surgical skillset and learning curve and is also related to the procedure and its associated complications, as well as the clinical condition of the patient population. It is important to note that the learning curve is not solely concerning the technical skills of the surgeon but also involves the knowledge, expertise and ability of the surgical staff in proper setup, docking and instrument handling [95,99]. As with every new technique and technology, it is natural for a surgical team to need time to become fully acquainted. A standardized training program should be established with simulator training to aid in the transition of efficiently adopting robotic procedures. It can be assumed that once the learning curve is overcome and completed, improved outcomes might be achieved, and the cost-effectiveness of the robot could overrule the standard laparoscopic approach [98]. Gray et al. reported that the surgeon’s experience in performing a robotic procedure has a direct correlation with the surgical outcomes of the procedure [100]. This highlights the importance of abiding by proper surgical training of specific techniques and procedures and completing the learning curve to efficiently perform robotic procedures. 

Recent years have witnessed a significant increase in revisional bariatric surgeries, as evidenced by a decade-long study involving 822 fellows in Fellowship Council (FC)-accredited programs from 2010 to 2019. The data reveal a noteworthy doubling of major revisional bariatric cases per fellow, indicating a discernible trend in the metabolic and bariatric surgery landscape. Interestingly, this surge is not uniform across all fellowship programs; rather, a select group of high-volume programs seems to be driving the increase in RBS procedures. This concentration underscores the importance of carefully choosing training programs, not only for exposure to primary MBS procedures but also for hands-on experience with revisional interventions. The study concludes that the growing prominence of RBS emphasizes the need for specialized training programs to adapt to the evolving dynamics of this field and facilitate the opportunity for residents and fellows to be properly exposed to the robotic platform and engage with it in a hands-on experience [101]. 

## 7. Conclusions

Long-term studies have demonstrated that up to 15% of patients require revisional bariatric surgery (RBS) due to weight-related issues or surgical complications, with expectations of increasing rates mirroring the increase in primary MBS procedures [2]. Several distinct surgical options exist and are dependent on the indication for revision, patient factors, surgeon experience and institution. Although RBS is often necessary and clinically indicated, it is important for surgeons to consider the potential increased risks associated with revision surgery. RBS has been reported to carry a higher rate of morbidity and reoperation compared to primary MBS [22]. Therefore, future studies are necessary to identify factors associated with increased risks of undesired outcomes following primary MBS procedures. 

The growing utilization of the robotic approach in bariatric surgery highlights the significance of fully understanding its role in RBS. Even though scarce evidence is currently available, published results are promising. Our review, in agreement with recently published systematic reviews and meta-analyses, revealed that the robotic approach in RBS is a feasible alternative to the popular laparoscopic approach despite a lack of evidence presenting a clear significant advantage. Further comprehensive research is required to fully investigate the variety in surgeon methodology, level of experience and surgical proficiency of the surgeon, surgical staff and institution and ascertain if these variables are significantly contributing to the rise in these distinctions. 

## Figures and Tables

**Table 1:** Open vs. laparoscopic approach of revisional bariatric surgery.

Study	Year Published	Operative Time (min)		Overall Postoperative Complication Rate (%)		Length of Stay (Days)		Reoperation Rate (%)	
		Open	Lap	Open	Lap	Open	Lap	Open	Lap
Shimizu et al. [83]	2013	-	-	39.4 *	15.7 *	9.5 *	5.4 *	-	-
Buchs et al. [6]	2014	250 *	270 *	10.7	14.3	9	8	25	19

Data are presented as frequency and percentage for categorical variables. Mean values of each variable were used. Lap: laparoscopic. * Represents statistically significant difference (*p* < 0.05).

**Table 2:** Laparoscopic vs. robotic approach of revisional bariatric surgery.

Study	Year Published	Operative Time (min)		Overall Postoperative Complication Rate (%)		Conversion to Open Surgery (%)		Length of Stay (Days)		Reoperation Rate (%)	
		Lap	Robotic	Lap	Robotic	Lap	Robotic	Lap	Robotic	Lap	Robotic
King et al. [85]	2021	-	-	5.2	1.9	-	-	62.6 h *	40.2 h *	-	-
Nasser et al. [86]	2020	For SG: 101.9 *	For SG: 145.2 *	For SG: 4.5 *	For SG: 6.7 *	For SG: 0.1	For SG: 0.3	For SG: 1.7 *	For SG: 1.9 *	For SG: 1.5 *	For SG: 2.4 *
		RYGB: 153.9 *	For RYGB: 196.7 *	For RYGB: 11.6 *	For RYGB: 9.3 *	For RYGB: 0.6	For RYGB: 0.7	For RYGB: 2.4	For RYGB: 2.4	For RYGB: 3.9	For RYGB: 3.8
Acevedo et al. [88]	2020	119.5 *	173.7 *	-	-	1.0	1.1	1.9 *	2.3 *	2.8 *	3.7 *
Buchs et al. [6]	2014	270 *	352 *	14.3	0	14.3	0	8	6	19	9.1
Beckmann et al. [87]	2020	167.6 *	130.7 *	22.2	7.3	-	-	6.2 *	4.9 *	11.1	2.4
Clapp et al. [89]	2019	103.7 *	167.7 *	No overall rate but found no significance between groups for each complication	No overall rate but found no significance between groups for each complication	-	-	1.7 *	2.3 *	-	-
Bertoni et al. [7]	2021	124	173	7.5	8.1	0.3	0.5	2.0	2.3	2.5	3.1
Moon et al. [91]	2020	113.3 *	155.5 *	-	-	-	-	2.0	2.5	0	3.3

Data are presented as frequency and percentage for categorical variables. Mean values of each variable were used. Lap: laparoscopic. SG: sleeve gastrectomy. RYGB: Roux-en-Y gastric bypass. * Represents statistically significant difference (*p* < 0.05).

## Data Availability

Not applicable.

## References

[1] Chang S.H., Stoll C.R., Song J., Varela J.E., Eagon C.J., Colditz G.A. (2014). The effectiveness and risks of bariatric surgery: An updated systematic review and meta-analysis, 2003–2012. JAMA Surg..

[2] Clapp B., Ponce J., Corbett J., Ghanem O.M., Kurian M., Rogers A.M., Peterson R.M., LaMasters T., English W.J. American Society for Metabolic and Bariatric Surgery 2022 estimate of metabolic and bariatric procedures performed in the United States. Surg. Obes. Relat. Dis..

[3] Oyefule O., Do T., Karthikayen R., Portela R., Abu Dayyeh B., McKenzie T., Kellogg T., Ghanem O.M. (2022). Secondary Bariatric Surgery-Does the Type of Index Procedure Affect Outcomes after Conversion?. J. Gastrointest. Surg..

[4] Clapp B., Harper B., Dodoo C., Klingsporn W., Barrientes A., Cutshall M., Tyroch A. (2020). Trends in revisional bariatric surgery using the MBSAQIP database 2015–2017. Surg. Obes. Relat. Dis..

[5] Khalaj A., Barzin M., Ebadinejad A., Mahdavi M., Ebrahimi N., Valizadeh M., Hosseinpanah F. (2023). Revisional Bariatric Surgery due to Complications: Indications and Outcomes. Obes. Surg..

[6] Buchs N.C., Pugin F., Azagury D.E., Huber O., Chassot G., Morel P. (2014). Robotic revisional bariatric surgery: A comparative study with laparoscopic and open surgery. Int. J. Med. Robot..

[7] Bertoni M.V., Marengo M., Garofalo F., Volontè F., La Regina D., Gass M., Mongelli F. (2021). Robotic-Assisted Versus Laparoscopic Revisional Bariatric Surgery: A Systematic Review and Meta-analysis on Perioperative Outcomes. Obes. Surg..

[8] Brethauer S.A., Kothari S., Sudan R., Williams B., English W.J., Brengman M., Kurian M., Hutter M., Stegemann L., Kallies K. (2014). Systematic review on reoperative bariatric surgery: American Society for Metabolic and Bariatric Surgery Revision Task Force. Surg. Obes. Relat. Dis..

[9] Iranmanesh P., Bajwa K.S., Felinski M.M., Shah S.K., Wilson E.B. (2020). Robotic Primary and Revisional Bariatric Surgery. Surg. Clin. N. Am..

[10] Vahibe A., Aizpuru M.J., Sarr M.G.M.F., Mundi M.S., A Vierkant R., Mckenzie T.M.F., Abu Dayyeh B.K., Ghanem O.M.M.F. (2023). Safety and Efficacy of Revisional Surgery as a Treatment for Malnutrition after Bariatric Surgery. J. Am. Coll. Surg..

[11] Dreifuss N.H., Mangano A., Hassan C., Masrur M.A. (2021). Robotic Revisional Bariatric Surgery: A High-Volume Center Experience. Obes. Surg..

[12] Wilson E.B., Sudan R. (2013). The evolution of robotic bariatric surgery. World J. Surg..

[13] Jung M.K., Hagen M.E., Buchs N.C., Buehler L.H., Morel P. (2017). Robotic bariatric surgery: A general review of the current status. Int. J. Med. Robot..

[14] Zhang Z., Miao L., Ren Z., Li Y. (2021). Robotic bariatric surgery for the obesity: A systematic review and meta-analysis. Surg. Endosc..

[15] Khorgami Z., Shoar S., Andalib A., Aminian A., Brethauer S.A., Schauer P.R. (2017). Trends in utilization of bariatric surgery, 2010–2014: Sleeve gastrectomy dominates. Surg. Obes. Relat. Dis..

[16] Kheirvari M., Nikroo N.D., Jaafarinejad H., Farsimadan M., Eshghjoo S., Hosseini S., Anbara T. (2020). The advantages and disadvantages of sleeve gastrectomy; clinical laboratory to bedside review. Heliyon.

[17] Lind R., Hage K., Ghanem M., Shah M., Vierkant R.A., Jawad M., Ghanem O.M., Teixeira A.F. (2023). Long-Term Outcomes of Sleeve Gastrectomy: Weight Recurrence and Surgical Non-responders. Obes. Surg..

[18] Diamantis T., Apostolou K.G., Alexandrou A., Griniatsos J., Felekouras E., Tsigris C. (2014). Review of long-term weight loss results after laparoscopic sleeve gastrectomy. Surg. Obes. Relat. Dis..

[19] Clapp B., Wynn M., Martyn C., Foster C., O’Dell M., Tyroch A. (2018). Long term (7 or more years) outcomes of the sleeve gastrectomy: A meta-analysis. Surg. Obes. Relat. Dis..

[20] Lauti M., Lemanu D., Zeng I.S.L., Su’a B., Hill A.G., MacCormick A.D. (2017). Definition determines weight regain outcomes after sleeve gastrectomy. Surg. Obes. Relat. Dis..

[21] Nedelcu M., Khwaja H.A., Rogula T.G. (2016). Weight regain after bariatric surgery-how should it be defined?. Surg. Obes. Relat. Dis..

[22] Lazzati A., Bechet S., Jouma S., Paolino L., Jung C. (2020). Revision surgery after sleeve gastrectomy: A nationwide study with 10 years of follow-up. Surg. Obes. Relat. Dis..

[23] Homan J., Betzel B., Aarts E.O., van Laarhoven K.J., Janssen I.M., Berends F.J. (2015). Secondary surgery after sleeve gastrectomy: Roux-en-Y gastric bypass or biliopancreatic diversion with duodenal switch. Surg. Obes. Relat. Dis..

[24] Weiner R.A., Weiner S., Pomhoff I., Jacobi C., Makarewicz W., Weigand G. (2007). Laparoscopic sleeve gastrectomy--influence of sleeve size and resected gastric volume. Obes. Surg..

[25] Obeidat F., Shanti H., Mismar A., Albsoul N., Al-Qudah M. (2015). The Magnitude of Antral Resection in Laparoscopic Sleeve Gastrectomy and its Relationship to Excess Weight Loss. Obes. Surg..

[26] Kehagias I., Spyropoulos C., Karamanakos S., Kalfarentzos F. (2013). Efficacy of sleeve gastrectomy as sole procedure in patients with clinically severe obesity (BMI ≤ 50 kg/m^2^). Surg. Obes. Relat. Dis..

[27] Keren D., Matter I., Lavy A. (2014). Lifestyle modification parallels to sleeve success. Obes. Surg..

[28] Lombardo M., Bellia A., Mattiuzzo F., Franchi A., Ferri C., Elvira P., Guglielmi V., D’Adamo M., Giuseppe A., Gentileschi P. (2015). Frequent follow-up visits reduce weight regain in long-term management after bariatric surgery. Bariatr. Surg. Pract. Patient Care..

[29] Felsenreich D.M., Langer F.B., Kefurt R., Panhofer P., Schermann M., Beckerhinn P., Sperker C., Prager G. (2016). Weight loss, weight regain, and conversions to Roux-en-Y gastric bypass: 10-year results of laparoscopic sleeve gastrectomy. Surg. Obes. Relat. Dis..

[30] Iannelli A., Schneck A.S., Noel P., Ben Amor I., Krawczykowski D., Gugenheim J. (2011). Re-sleeve gastrectomy for failed laparoscopic sleeve gastrectomy: A feasibility study. Obes Surg..

[31] Naik R.D., Choksi Y.A., Vaezi M.F. (2016). Consequences of bariatric surgery on oesophageal function in health and disease. Nat. Rev. Gastroenterol. Hepatol..

[32] Popescu A.L., Ioniţa-Radu F., Jinga M., Gavrilă A.I., Săvulescu F.A., Fierbinţeanu-Braticevici C. (2018). Laparoscopic sleeve gastrectomy and gastroesophageal reflux. Rom. J. Intern. Med..

[33] Casillas R.A., Um S.S., Zelada Getty J.L., Sachs S., Kim B.B. (2016). Revision of primary sleeve gastrectomy to Roux-en-Y gastric bypass: Indications and outcomes from a high-volume center. Surg. Obes. Relat. Dis..

[34] Li S., Jiao S., Zhang S., Zhou J. (2021). Revisional Surgeries of Laparoscopic Sleeve Gastrectomy. Diabetes Metab. Syndr. Obes..

[35] Rebibo L., Hakim S., Dhahri A., Yzet T., Delcenserie R., Regimbeau J.M. (2016). Gastric Stenosis After Laparoscopic Sleeve Gastrectomy: Diagnosis and Management. Obes. Surg..

[36] Parikh A., Alley J.B., Peterson R.M., Harnisch M.C., Pfluke J.M., Tapper D.M., Fenton S.J. (2012). Management options for symptomatic stenosis after laparoscopic vertical sleeve gastrectomy in the morbidly obese. Surg. Endosc..

[37] Burgos A.M., Csendes A., Braghetto I. (2013). Gastric stenosis after laparoscopic sleeve gastrectomy in morbidly obese patients. Obes. Surg..

[38] Vilallonga R., Himpens J., van de Vrande S. (2013). Laparoscopic management of persistent strictures after laparoscopic sleeve gastrectomy. Obes. Surg..

[39] Nedelcu M., Manos T., Cotirlet A., Noel P., Gagner M. (2015). Outcome of leaks after sleeve gastrectomy based on a new algorithm adressing leak size and gastric stenosis. Obes. Surg..

[40] Márquez M.F., Ayza M.F., Lozano R.B., Morales Mdel M., Díez J.M., Poujoulet R.B. (2010). Gastric leak after laparoscopic sleeve gastrectomy. Obes. Surg..

[41] Ghanem O.M., Abu Dayyeh B.K., Kellogg T.A. (2017). Management of Gastropleural Fistula after Revisional Bariatric Surgery: A Hybrid Laparoendoscopic Approach. Obes. Surg..

[42] Süsstrunk J., Wartmann L., Mattiello D., Köstler T., Zingg U. (2021). Incidence and Prognostic Factors for the Development of Symptomatic and Asymptomatic Marginal Ulcers After Roux-en-Y Gastric Bypass Procedures. Obes. Surg..

[43] Abboud D.M., Yao R., Rapaka B., Ghazi R., Ghanem O.M., Abu Dayyeh B.K. (2022). Endoscopic Management of Weight Recurrence Following Bariatric Surgery. Front. Endocrinol..

[44] Di Palma A., Liu B., Maeda A., Anvari M., Jackson T., Okrainec A. (2021). Marginal ulceration following Roux-en-Y gastric bypass: Risk factors for ulcer development, recurrence and need for revisional surgery. Surg. Endosc..

[45] Coblijn U.K., Goucham A.B., Lagarde S.M., Kuiken S.D., van Wagensveld B.A. (2014). Development of ulcer disease after Roux-en-Y gastric bypass, incidence, risk factors, and patient presentation: A systematic review. Obes. Surg..

[46] Beran A., Shaear M., Al-Mudares S., Sharma I., Matar R., Al-Haddad M., Salame M., Portela R., Clapp B., Abu Dayyeh B.K. (2023). Predictors of marginal ulcer after gastric bypass: A systematic review and meta-analysis. J. Gastrointest. Surg..

[47] Chung W.C., Jeon E.J., Lee K.-M., Paik C.N., Jung S.H., Oh J.H., Kim J.H., Jun K.-H., Chin H.M. (2012). Incidence and clinical features of endoscopic ulcers developing after gastrectomy. World J. Gastroenterol..

[48] Adduci A.J., Phillips C.H., Harvin H. (2016). Prospective diagnosis of marginal ulceration following Roux-en-Y gastric bypass with computed tomography. Radiol. Case Rep..

[49] Bacoeur-Ouzillou O., Perinel J., Pelascini E., Abdallah M., Poncet G., Pasquer A., Robert M. (2022). Management strategies of anastomotic ulcer after gastric bypass and risk factors of recurrence. Surg. Endosc..

[50] Chang P.C., Huang C.K., Tai C.M., Huang I.Y., Hsin M.C., Hung C.M. (2017). Revision using totally hand-sewn gastrojejunostomy and truncal vagotomy for refractory marginal ulcer after laparoscopic Roux-en-y gastric bypass: A case series. Surg. Obes. Relat. Dis..

[51] Khrucharoen U., Weitzner Z.N., Chen Y., Dutson E.P. (2022). Incidence and risk factors for early gastrojejunostomy anastomotic stricture requiring endoscopic intervention following laparoscopic Roux-en-Y gastric bypass: A MBSAQIP analysis. Surg. Endosc..

[52] Csendes A., Burgos A.M., Burdiles P. (2009). Incidence of anastomotic strictures after gastric bypass: A prospective consecutive routine endoscopic study 1 month and 17 months after surgery in 441 patients with morbid obesity. Obes. Surg..

[53] Yimcharoen P., Heneghan H., Chand B., Talarico J.A., Tariq N., Kroh M., Brethauer S.A. (2012). Successful management of gastrojejunal strictures after gastric bypass: Is timing important?. Surg. Obes. Relat. Dis..

[54] Almby K., Edholm D. (2019). Anastomotic Strictures After Roux-en-Y Gastric Bypass: A Cohort Study from the Scandinavian Obesity Surgery Registry. Obes. Surg..

[55] Palermo M., Acquafresca P.A., Rogula T., Duza G.E., Serra E. (2015). Late surgical complications after gastric by-pass: A literature review. Arq. Bras. Cir. Dig..

[56] Shariq O.A.M., Portela R., Bews K.A.B., Mundi M.S., Kellogg T., Habermann E.B., Abu Dayyeh B., Kendrick M.L., Ghanem O.M. (2023). Impact of Early Gastrojejunal Stenosis on Weight Loss in Patients Undergoing Roux-en-Y Gastric Bypass. Surg. Laparosc. Endosc. Percutaneous Tech..

[57] Lois A.W., Frelich M.J., Goldblatt M.I., Wallace J.R., Gould J.C. (2015). Gastrojejunostomy technique and anastomotic complications in laparoscopic gastric bypass. Surg. Obes. Relat. Dis..

[58] Edholm D. (2019). Systematic Review and Meta-analysis of Circular- and Linear-Stapled Gastro-jejunostomy in Laparoscopic Roux-en-Y Gastric Bypass. Obes. Surg..

[59] Fakas S., Elias M., Lim D., Meytes V. (2021). Comparison of gastrojejunostomy techniques and anastomotic complications: A systematic literature review. Surg. Endosc..

[60] Mahmoud T., Beran A., Bazerbachi F., Matar R., Jaruvongvanich V., Razzak F.A., Abboud D.M., Vargas E.J., Martin J.A., Kellogg T.A. (2023). Lumen-apposing metal stents for the treatment of benign gastrointestinal tract strictures: A single-center experience and proposed treatment algorithm. Surg. Endosc..

[61] King W.C., Hinerman A.S., Belle S.H., Wahed A.S., Courcoulas A.P. (2018). Comparison of the Performance of Common Measures of Weight Regain After Bariatric Surgery for Association with Clinical Outcomes. JAMA.

[62] Athanasiadis D.I., Martin A., Kapsampelis P., Monfared S., Stefanidis D. (2021). Factors associated with weight regain post-bariatric surgery: A systematic review. Surg. Endosc..

[63] Cooper T.C., Simmons E.B., Webb K., Burns J.L., Kushner R.F. (2015). Trends in Weight Regain Following Roux-en-Y Gastric Bypass (RYGB) Bariatric Surgery. Obes. Surg..

[64] Hutch C.R., Sandoval D. (2017). The Role of GLP-1 in the Metabolic Success of Bariatric Surgery. Endocrinology.

[65] Abu Dayyeh B., Portela R., Mahmoud T., Ghazi R., Ghanem O.M. (2022). A novel approach for weight regain after Roux-en-Y gastric bypass: Staged transoral outlet reduction (TORe) followed by surgical type 1 distalization. VideoGIE.

[66] Giannopoulos S., Kapsampelis P., Pokala B., Connors J.D.N., Hilgendorf W., Timsina L., Clapp B., Ghanem O., Kindel T.L., Stefanidis D. (2023). Bariatric Surgeon Perspective on Revisional Bariatric Surgery (RBS) for Weight Recurrence. Surg. Obes. Relat. Dis..

[67] Mahawar K.K., Graham Y., Carr W.R.J., Jennings N., Schroeder N., Balupuri S., Small P.K. (2015). Revisional Roux-en-Y Gastric Bypass and Sleeve Gastrectomy: A Systematic Review of Comparative Outcomes with Respective Primary Procedures. Obes. Surg..

[68] Kermansaravi M., Davarpanah Jazi A.H., Shahabi Shahmiri S., Eghbali F., Valizadeh R., Rezvani M. (2021). Revision procedures after initial Roux-en-Y gastric bypass, treatment of weight regain: A systematic review and meta-analysis. Updates Surg..

[69] Franken R.J., Franken J., Sluiter N.R., de Vries R., Euser S., Gerdes V.E.A., de Brauw M. (2023). Efficacy and safety of revisional treatments for weight regain or insufficient weight loss after Roux-en-Y gastric bypass: A systematic review and meta-analysis. Obes. Rev..

[70] Lind R., Ghanem O.M., Ghanem M., Teixeira A.F., Jawad M.A. (2022). Duodenal Switch Conversion in Non-responders or Weight Recurrence Patients. Obes. Surg..

[71] Eisenberg D., Azagury D.E., Ghiassi S., Grover B.T., Kim J.J. (2017). AkingSMBS Position Statement on Postprandial Hyperinsulinemic Hypoglycemia after Bariatric Surgery. Surg. Obes. Relat. Dis..

[72] Salehi M., Vella A., McLaughlin T., Patti M.E. (2018). Hypoglycemia After Gastric Bypass Surgery: Current Concepts and Controversies. J. Clin. Endocrinol. Metab..

[73] Xu Q., Zou X., You L., Wu W., Zhu H., Wang L., Yuan T., Zhao Y. (2021). Surgical Treatment for Postprandial Hypoglycemia After Roux-en-Y Gastric Bypass: A Literature Review. Obes. Surg..

[74] Pędziwiatr M., Małczak P., Wierdak M., Rubinkiewicz M., Pisarska M., Major P., Wysocki M., Karcz W., Budzyński A. (2018). Revisional Gastric Bypass Is Inferior to Primary Gastric Bypass in Terms of Short- and Long-term Outcomes-Systematic Review and Meta-Analysis. Obes. Surg..

[75] Zhang L., Tan W.H., Chang R., Eagon J.C. (2015). Perioperative risk and complications of revisional bariatric surgery compared to primary Roux-en-Y gastric bypass. Surg. Endosc..

[76] Vallois A., Menahem B., Le Roux Y., Bion A.L., Meunier H., Gautier T., Contival N., Mulliri A., Lubrano J., Parienti J.-J. (2019). Revisional Roux-en-Y Gastric Bypass: A Safe Surgical Opportunity? Results of a Case-Matched Study. Obes. Surg..

[77] Iranmanesh P., Fam J., Nguyen T., Talarico D., Chandwani K.D., Bajwa K.S., Felinski M.M., Katz L.V., Mehta S.S., Myers S.R. (2021). Outcomes of primary versus revisional robotically assisted laparoscopic Roux-en-Y gastric bypass: A multicenter analysis of ten-year experience. Surg. Endosc..

[78] Bennett W.C., Garbarine I.C., Mostellar M., Lipman J., Sanchez-Casalongue M., Farrell T., Zhou R. (2023). Comparison of early post-operative complications in primary and revisional laparoscopic sleeve gastrectomy, gastric bypass, and duodenal switch MBSAQIP-reported cases from 2015 to 2019. Surg Endosc..

[79] Snyder B.E., Wilson T., Scarborough T., Yu S., Wilson E.B. (2008). Lowering gastrointestinal leak rates: A comparative analysis of robotic and laparoscopic gastric bypass. J. Robot. Surg..

[80] Buchs N.C., Morel P., Azagury D.E., Jung M., Chassot G., Huber O., Hagen M.E., Pugin F. (2014). Laparoscopic versus robotic Roux-en-Y gastric bypass: Lessons and long-term follow-up learned from a large prospective monocentric study. Obes. Surg..

[81] Rogula T., Koprivanac M., Janik M.R., Petrosky J.A., Nowacki A.S., Dombrowska A., Kroh M., Brethauer S., Aminian A., Schauer P. (2018). Does Robotic Roux-en-Y Gastric Bypass Provide Outcome Advantages over Standard Laparoscopic Approaches?. Obes. Surg..

[82] Sebastian R., Howell M.H., Chang K.-H., Adrales G., Magnuson T., Schweitzer M., Nguyen H. (2019). Robot-assisted versus laparoscopic Roux-en-Y gastric bypass and sleeve gastrectomy: A propensity score-matched comparative analysis using the 2015–2016 MBSAQIP database. Surg Endosc..

[83] Shimizu H., Annaberdyev S., Motamarry I., Kroh M., Schauer P.R., Brethauer S.A. (2013). Revisional bariatric surgery for unsuccessful weight loss and complications. Obes. Surg..

[84] Van Koughnett J.A., Jayaraman S., Eagleson R., Quan D., van Wynsberghe A., Schlachta C.M. (2009). Are there advantages to robotic-assisted surgery over laparoscopy from the surgeon’s perspective?. J. Robot. Surg..

[85] King K., Galvez A., Stoltzfus J., Claros L., El Chaar M. (2021). Robotic-Assisted Surgery Results in a Shorter Hospital Stay Following Revisional Bariatric Surgery. Obes. Surg..

[86] Nasser H., Munie S., Kindel T.L., Gould J.C., Higgins R.M. (2020). Comparative analysis of robotic versus laparoscopic revisional bariatric surgery: Perioperative outcomes from the MBSAQIP database. Surg. Obes. Relat. Dis..

[87] Beckmann J.H., Mehdorn A.-S., Kersebaum J.-N., von Schönfels W., Taivankhuu T., Laudes M., Egberts J.-H., Becker T. (2020). Pros and Cons of Robotic Revisional Bariatric Surgery. Visc. Med..

[88] Acevedo E., Mazzei M., Zhao H., Lu X., Edwards M.A. (2020). Outcomes in conventional laparoscopic versus robotic-assisted revisional bariatric surgery: A retrospective, case-controlled study of the MBSAQIP database. Surg. Endosc..

[89] Clapp B., Liggett E., Jones R., Lodeiro C., Dodoo C., Tyroch A. (2019). Comparison of robotic revisional weight loss surgery and laparoscopic revisional weight loss surgery using the MBSAQIP database. Surg. Obes. Relat. Dis..

[90] Moon R.C., Segura A.R., Teixeira A.F., Jawad M.A. (2020). Feasibility and safety of robot-assisted bariatric conversions and revisions. Surg. Obes. Relat. Dis..

[91] Snyder B., Wilson T., Woodruff V., Wilson E. (2013). Robotically assisted revision of bariatric surgeries is safe and effective to achieve further weight loss. World J. Surg..

[92] Schauer P., Ikramuddin S., Hamad G., Gourash W. (2003). The learning curve for laparoscopic Roux-en-Y gastric bypass is 100 cases. Surg. Endosc..

[93] Oliak D., Ballantyne G.H., Weber P., Wasielewski A., Davies R.J., Schmidt H.J. (2003). Laparoscopic Roux-en-Y gastric bypass: Defining the learning curve. Surg. Endosc..

[94] Buchs N.C., Pugin F., Bucher P., Hagen M.E., Chassot G., Koutny-Fong P., Morel P. (2012). Learning curve for robot-assisted Roux-en-Y gastric bypass. Surg. Endosc..

[95] Vilallonga R., Fort J.M., Gonzalez O., Caubet E., Boleko A., Neff K.J., Armengol M. (2012). The Initial Learning Curve for Robot-Assisted Sleeve Gastrectomy: A Surgeon’s Experience While Introducing the Robotic Technology in a Bariatric Surgery Department. Minim. Invasive Surg..

[96] Romero R.J., Kosanovic R., Rabaza J.R., Seetharamaiah R., Donkor C., Gallas M., Gonzalez A.M. (2013). Robotic sleeve gastrectomy: Experience of 134 cases and comparison with a systematic review of the laparoscopic approach. Obes. Surg..

[97] Abdelgawad M., De Angelis F., Iossa A., Rizzello M., Cavallaro G., Silecchia G. (2016). Management of Complications and Outcomes After Revisional Bariatric Surgery: 3-Year Experience at a Bariatric Center of Excellence. Obes. Surg..

[98] Li K., Zou J., Tang J., Di J., Han X., Zhang P. (2016). Robotic Versus Laparoscopic Bariatric Surgery: A Systematic Review and Meta-Analysis. Obes. Surg..

[99] Vanetta C., Dreifuss N.H., Schlottmann F., Mangano A., Cubisino A., Valle V., Baz C., Bianco F.M., Hassan C., Gangemi A. (2022). Current Status of Robot-Assisted Revisional Bariatric Surgery. J. Clin. Med..

[100] Gray K.D., Moore M.D., Elmously A., Bellorin O., Zarnegar R., Dakin G., Pomp A., Afaneh C. (2018). Perioperative Outcomes of Laparoscopic and Robotic Revisional Bariatric Surgery in a Complex Patient Population. Obes. Surg..

[101] Monfared S., Weis J.J., Shah S.K., Scott D.J., Felinski M.M., Wilson E.B. (2023). The rising tide of revisional surgery: Tracking changes in index cases among bariatric-accredited fellowships. Surg. Endosc..

